# Incivility, bullying, and poor health and well-being among students: a Swedish national study in higher education institutions

**DOI:** 10.3389/fpubh.2024.1400520

**Published:** 2024-10-30

**Authors:** Aziz Mensah, Susanna Toivanen, Christina Björklund

**Affiliations:** ^1^School of Health, Care, and Social Welfare, Mälardalen University, Västerås, Sweden; ^2^Unit of Intervention and Implementation Research for Worker Health, Institute for Environmental Medicine Karolinska Institutet, Stockholm, Sweden

**Keywords:** incivility, bullying, health and well-being, gender, mediation, moderation, structural equation model, students

## Abstract

**Objective:**

Exposure to incivility and bullying among students in higher education institutions may have detrimental health and well-being outcomes. Nevertheless, the mechanism and interconnected pathways through which incivility and bullying are linked with poor health and well-being remain largely unexplored. The aim of this study is to investigate the relationships between incivility, bullying, and poor health and well-being among students in higher education institutions in Sweden, and whether gender influences these relationships. Furthermore, we examine whether bullying plays a mediating role in the relationship between incivility and poor health and well-being.

**Methods:**

We analyzed a cross-sectional dataset of students drawn from 38 universities that are members of the association of Swedish higher education institutions. The data were collected from May to July 2021, covering 11,162 women and 6,496 men. Confirmatory factor analysis and structural equation modeling (SEM) were utilized to estimate the relationships between incivility, bullying, and poor health and well-being. Additionally, multigroup analysis was applied to estimate the interactive effect of gender in these relationships.

**Results:**

Reports of both incivility and bullying were more prevalent among women than men. The results showed that incivility had direct relationships with both bullying $\left(\beta =0.578,p<0.01\right)$ and poor health and well-being $\left(\beta =0.301,p<0.01\right)$ . However, the relationship between bullying and poor health and well-being was not significant. There were statistically significant gender differences in the relationships between incivility, bullying, and poor health and well-being ( $\Delta \chi^{2}\left(23\right)=179.18,p<0.01)$ . Nevertheless, bullying did not significantly mediate the relationship between incivility and poor health and well-being.

**Conclusion:**

The current study demonstrates that governments, university authorities, and policymakers must consider gender differences in incivility and bullying when developing policies and interventions intended to reduce these kinds of behaviors in organizations.

## 1 Introduction

The last three decades have seen increasing scholarship on covert, subtle, and nonphysical demonstrations of interpersonal harm, such as incivility (1). Incivility is the most common form of interpersonal mistreatment in organizations (2). For instance, findings from previous studies conducted in various organizations in the US, Canada, and the UK suggest that about 20% of workers experience incivility (3–5). Years after these studies, evidence from systematic reviews found a high prevalence of incivility ranging from 55 to 90% among employees in organizations (2, 6). Similarly, a study conducted in Sweden by Torkelson et al. (7) reported an incivility rate of 73% among faculty members in higher education institutions (HEIs).

Even though HEIs are where students receive training to improve their knowledge, manners, and social responsibility, the prevalence of incivility among students in HEIs may not be different from that among workers. For example, a study conducted in HEIs by Wagner et al. (8) found a high prevalence of incivility (65.22%) in the form of using electronic devices during class sessions for unrelated learning purposes among students in the US. Similarly, Muliira et al. (9) demonstrated a high prevalence (40–88%) of various forms of uncivil behavior among nursing students in a higher education institution in Oman. One recent study that was conducted in Sweden during the COVID-19 pandemic in 2021 indicated that about 52% of students experienced incivility in the form of being interrupted or spoken over (10). The study further stated that women reported more incivility than men in HEIs in Sweden. Nevertheless, the literature on incivility among students in HEIs in Sweden is scant. Despite the evidence for prevalent incivility, the policies, regulations, and codes of conduct that address interpersonal mistreatment at HEIs have not been able to isolate and counter uncivil behavior in a thorough manner (11). The Swedish higher education ordinance, which seeks to address interpersonal mistreatment at HEIs (12), has paid less attention to incivility as compared to bullying, harassment, and violence. Meanwhile, incivility has been identified as having negative health (3, 13) and behavioral consequences, such as bullying (14, 15).

### 1.1 Incivility and health and well-being

According to Andersson and Pearson, incivility is defined as “low-intensity deviant behavior with ambiguous intent to harm the target, in violation of workplace norms for mutual respect. Uncivil behaviors are characteristically rude and discourteous, displaying a lack of regard for others” (16). The authors described this concept as a particular kind of deviance (17), which may consequently represent a subset of antisocial employee conduct (3). Additionally, they posited that instigators of uncivil behavior are mostly ignorant of the act. In expanding the debate on the sources of incivility, Clark (18, 19) argued that aside from the workplace, incivility may surface in other environments, such as HEIs. According to Clark, incivility is a behavior “demonstrated by students or faculty… [that] violates the norms of mutual respect in the teaching-learning environment” (18). Clark (19) explained that incivility encompass a continuum of behaviors, from minor to severe offenses.

Several fundamental elements differentiate the incivility from other forms of interpersonal mistreatment and negative behavior constructs, such as abusive supervision, harassment, and bullying. For example, unlike other forms of negative behavior, incivility has only a low intensity and an ambiguous intent to harm. Furthermore, unlike the overt nature of other forms of negative workplace behavior, the covert nature of incivility makes the act difficult to discern and makes it very challenging for the victim to assign the action to the instigator. Additionally, incivility, as a concept, is broader than other negative behaviors, such as abusive supervision. This is because unlike abusive supervision, which may be primarily instigated by someone with higher status in the organization (e.g., a supervisor or teacher), uncivil behavior may be instigated by anyone regardless of their status or social identity (e.g., faculty members and students).

In an attempt to provide a better understanding of the relationship between incivility and negative health outcomes, Cortina et al. (3) argued that the theoretical “snow-balling effect” of the link between uncivil behavior and negative health and organizational effects is similar to the model of daily hassles derived from stress and coping theories (20). Daily hassles are minor daily experiences that are appraised as threatening and may consequently affect the well-being of the person (20). Using the stress and coping theory, many studies have demonstrated that although incivility is a covert and subtle form of violence, it may have a detrimental effect on both physical and mental health outcomes. For example, past research has found relationships between incivility and psychological distress (3, 14, 21), psychological ill-being and well-being (13, 22), burnout, emotional exhaustion, depression, stress, anxiety, physical health, and job satisfaction (19, 23). However, a major limitation of the literature is that most of these studies have been based on working populations, which may have different antecedents of incivility than students in higher education. Moreover, even though the few studies that have examined the relationship between incivility and detrimental health outcomes among students in higher education have found positive associations (24–26), these studies were based outside of Sweden. This makes us sceptical about the strength, magnitude, and direction of the association for students in HEIs in Sweden. Thus, there is a need to expand our knowledge about incivility and its association with negative health outcomes among students in HEIs in Sweden in order to develop effective preventive strategies and interventions. To do so, we posit the following research hypotheses:

*H1*: Incivility is positively associated with poor health and well-being.

### 1.2 The mediating role of bullying

Bullying is a form of interpersonal mistreatment in organisations. Although the question of what behaviors constitute bullying remains controversial, one of the widely accepted definitions of bullying among students was given by Olweus. According to that author, “a student is being bullied or victimised when he or she is exposed repeatedly and over time to negative actions on the part of one or more other students” (27). A remarkable prevalence on the part of bullying (5–70%), has been found in educational institutions, although results have been inconsistent (28, 29).

A report compiled by the American Association of Nurses to address incivility, bullying, and violence in organizations stated that the failure to resolve incivility may result in bullying (30). Similarly, but without empirical evidence, Andersson and Pearson (16) alluded to the fact that despite the low intensity of incivility and its ambiguous intent to harm, its continuous occurrence may develop into aggressive and intense behavior if left unchecked. The authors explained that when negative social behavior continuously occurs, it ultimately reaches a tipping point at which it is seen as purposeful aggressiveness rather than unintended or ambiguous behavior (16). In light of this, studies have found a relationship between incivility and bullying (14, 15). To date, Holm et al. (14) is the only empirical study that has provided evidence of a positive relationship between incivility and bullying in Sweden. However, in contrast to our study, their study design was based on working populations. Conducting more empirical studies will provide more information on the relationship between incivility and bullying in order to improve existing policies and interventions in HEIs in Sweden.

On the other hand, bullying has been found to be associated with negative health outcomes, such as depression and burnout (31), anxiety, poor general health (32), poor mental health (33), and suicidal ideation (34). Collectively, the theoretical and empirical arguments advanced thus far suggest that incivility may have a direct association with bullying and that bullying may, in turn, have a direct association with poor health outcomes. Thus, it seems plausible that bullying may mediate the relationship between incivility and negative health outcomes among students in HEIs. However, this mediating effect on the part of bullying is unexplored. Investigating its role and underlying mechanism and process is a good start to providing knowledge about the nomological network among incivility, bullying, and negative health outcomes. Thus, we post the following hypotheses:

*H2*: Incivility is positively associated with bullying.

*H3*: Bullying is positively associated with poor health and well-being.

*H4*: Bullying mediates the relationship between incivility and poor health and well-being.

### 1.3 The moderating role of gender

Studies on gender differences have suggested that gender stereotypes may influence the threshold that men and women adopt in perceiving (35) and reacting to (36) similar negative behaviors like incivility and bullying that they encounter during their daily lives. The social role theory, which was proposed by Eagly (37), is widely used to explain gender differences in society. The theory posits that society’s division of roles based on gender has led to commonly held gender stereotypes that often attribute agency-related traits (e.g., power, aggressiveness, competence, and independence) to men and communion-related traits (e.g., nurturing, friendliness, interdependence, caring, expressiveness, compassion, and cooperativeness) to women. Eagly (37) explained that both men and women tend to behave in conformity with their expected and prescribed gendered norms, as described by societal and cultural standards. Meanwhile, whereas the communal traits of women are more related to civil behavior, the agentic traits (e.g., power and aggressiveness) of men highlight the tendency for uncivil behavior and bullying on the part of men (38, 39). Barnett et al. (40) established that on average, men are more prone to committing minor legal and moral violations than women.

In view of this, the findings of previous research have indicated a gender difference in incivility and bullying, with women more often having been targets of incivility and bullying than men (1, 38, 39, 41, 42). Furthermore, the few studies that have examined gender differences in incivility and bullying have identified their influence on poor physical and mental health (13, 43–45), suggesting that gender may play a pivotal role in the relationships between incivility, bullying, and negative health outcomes.

### 1.4 The present study

Although the relationships between incivility and poor health and well-being among workers-to-workers, students-to-faculty, and faculty-to-students are well known (3, 13, 22–26), studies on incivility from a combined direction on students in HEIs, particularly in Sweden, are less common. To the best of our knowledge, no studies have explored the relationship between incivility and bullying among students in HEIs in Sweden. Also, in spite of the gender difference in the prevalence of incivility and bullying in HEIs (10), studies that investigate the relationships between incivility, bullying, and poor health and well-being outcomes have often ignored the role of gender (24, 25, 46). As far as we know, this is the first study to examine the mediating effect of bullying in the relationship between incivility and poor health and well-being.

**Figure 1:**
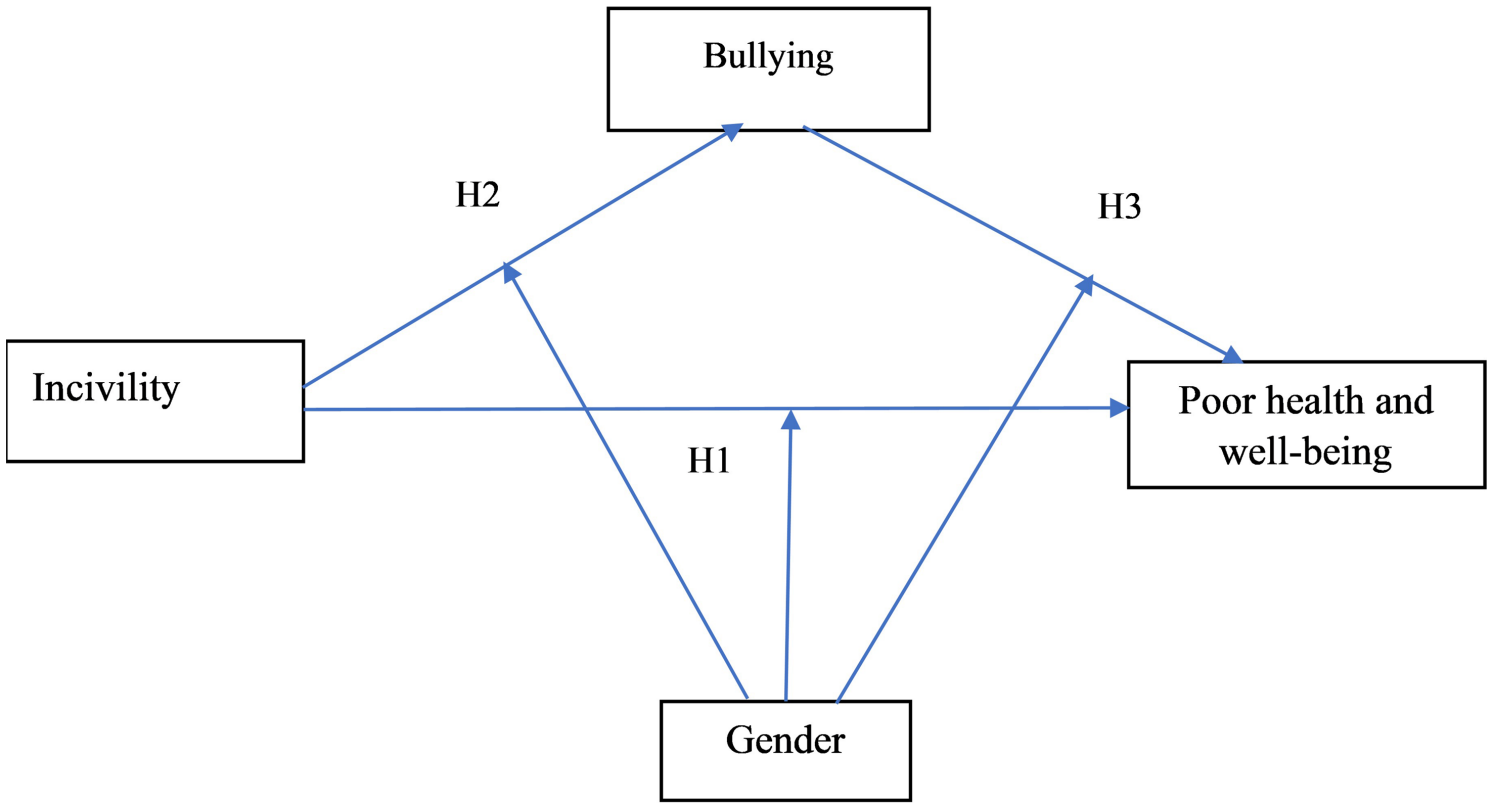
Conceptual framework for the study.

The aim of the current research is to examine the relationships between incivility, bullying, and poor health and well-being among students in HEIs in Sweden, and how gender influences these relationships. Furthermore, the study investigates the mediating role of bullying in the relationship between incivility and poor health and well-being among students in HEIs. Figure 1 shows the conceptual framework for our study.

## 2 Methods

### 2.1 Study participants and procedure

This study was based on survey data conducted at 38 universities that are part of the Association of Swedish Higher Education Institutions (SUHF). The data were collected from March to July 2021 by Statistics Sweden and included students, PhD students, and faculty members (employees). The survey was mainly designed by researchers at Karolinska University and Gothenburg University, and further discussed with other researchers from different universities who were part of the collaboration (10). The sample consisted of students, PhD students, and faculty members who had been in the university since the autumn of 2020. Although the survey included all students who were in smaller universities, the sampling for larger universities was based on the optimal allocation for each subpopulation. The details of the sampling technique and sampling design are provided elsewhere (10). After series of follow-ups, a total of 38,918 students and faculty members participated in the survey. We excluded PhD students and faculty members, limiting the study to only bachelor’s and master’s students, and obtained 18,582 participants. After processing the data, only 17,658 students were included in our study. We obtained ethical approval for the study from the Swedish Ethical Review board (Reference numbers: 2020-03499; 2021-06509-02).

### 2.2 Measures

#### 2.2.1 Poor health and well-being

Health and well-being were measured with the third version of the Copenhagen Psycho-Social Questionnaire (COPSOQ III). In the COPSOQS III, health and well-being were based on the General Health Questionnaire (GHQ) (47). The GHQ consists of 12-items covering general health and different well-being outcomes. However, the current study was limited to a short version of the GHQ, with eight items subdivided into four main factors as follows: (1) stress (e.g., “Have you had problems relaxing?”), (2) burnout (e.g., “Have you felt worn out?”), (3) self-reported health (“In general, would you say your health is…?”), and (4) intention to leave (e.g., “Have you considered resigning from your current studies?”). The responses to all the items were measured using a Likert scale from 1 to 5. We recoded the responses in descending order so that respondents with higher scores would have poorer health and well-being. Furthermore, as in the study conducted by Shahidi et al. (48), we transformed the scale for each item so as to make it 0 to 100. Previous studies have identified the scale to have good internal consistency of reliability, with a Cronbach alpha of 0.72 to 0.85 (49). The GHQ is a widely used questionnaire that has been validated among populations in Sweden (49).

#### 2.2.2 Bullying

A one-item questionnaire from the COPSOQ-III instrument was used to measure bullying. The scale was then adapted to suit a higher education setting. The students were asked, “During the last 12 months, have you been exposed to bullying at your place of study?” Responses ranged from 1 (yes, daily) to 5 (no) on a Likert scale. Because the scale decreases as bullying increases, we recoded the scale in increasing order so that people with higher levels of bullying would be assigned higher scores. The scale has been validated among populations in Sweden (50).

#### 2.2.3 Incivility

Incivility was assessed with a 12-item questionnaire that was developed by Cortina (11). The scale was then adapted to suit a higher education setting. In the questionnaire, the students were asked whether they had experienced the following situations with their teachers or fellow students in the last 12 months: e.g., “Have they paid little attention to your statements or showed little interest in your opinions?” The responses ranged from 1 (never) to 5 (many times) on a Likert scale. This instrument has been identified as having an excellent internal consistency of reliability, with a Cronbach alpha of 0.92 (11). This measuring instrument has been widely used and validated in working populations (11) and in higher education settings (13, 51).

#### 2.2.4 Covariates

Age, gender (men and women), place of birth (born in Sweden and born outside of Sweden), subject of study (sciences, engineering sciences, medicine and health sciences, agricultural and veterinary sciences, social sciences, and humanities and arts), were considered as potential covariates in the study (14, 18, 21) to ensure that the estimated results are due to the variable of interest and not confounding factors.

### 2.3 Statistical analyses

The mean, standard deviation, and prevalence were estimated to understand the general distribution and characteristics of the data by gender. Afterward, several analytical strategies were used. First, confirmatory factor analysis (CFA) was adopted to estimate the measurement model or latent constructs (Model 1). Confirmatory factor analysis was chosen because the scales with multiple indicators in our study (i.e., incivility and poor health and well-being) had been previously tested on various groups, samples, populations, and locations (13, 50, 51). Thus, theoretical and empirical evidence was available. However, because applying standard scales or translating and adjusting the original scale for diverse populations and samples may affect the performance of the scale (52), we assessed the reliability, convergent validity, and discriminant validity of the latent constructs before investigating our hypotheses. As recommended by Bagozzi (53), several methods were used to assess the reliability, convergent validity, and discriminant validity of the latent constructs. More specifically, in the current study, the reliability of the measurement models was assessed using (1) the Cronbach’s alpha coefficient and (2) the composite reliability index. Also, the convergent validity of the constructs was assessed using (1) the standardised factor loadings of the items and (2) the average variance extracted (AVE). Furthermore, discriminant validity was tested using (1) the comparison of AVE and maximum shared square variance (MSV) and (2) the comparison of the square root of AVE and correlation of the constructs. We relied on Cheung et al.’s (52) cut-off criteria for the reliability and validity of the latent constructs.

Second, structural equation modeling (SEM) with a maximum likelihood estimation (MLE) was adopted to estimate the relationships between incivility, bullying, and poor health and well-being (Model 2). SEM is a strong analytical tool for revealing the intricate mechanisms and interrelationships between multiple factors (54). Unlike traditional regressions (e.g., linear regression, logistic regression, Poisson regression, etc.), SEM allows the estimation of measurement errors of the latent constructs, which is important in computing accurate and reliable estimates. In this study, SEM was fitted as an extension of the CFA. This was performed by, first, extending direct paths from incivility to bullying (*path a*), from bullying to poor health and well-being (*path b*), and from incivility to poor health and well-being (*path c*). Second, the indirect effect of bullying was estimated by multiplying *paths a* and *b* (*path ab*). Because the bootstrapping procedure provides more accurate results when estimating the statistical significance of an indirect effect (55, 56), a 5,000-iteration bootstrapping procedure was applied to estimate the bias-corrected 95% confidence interval (CI) (55, 57). All estimated paths from the SEM were adjusted for covariates and standardised for easy interpretation.

Third, a multigroup analysis was performed to investigate the gender differences in the relationships between incivility, bullying, and poor health and well-being. Here, two competing models were fitted to the data: the constrained model and the unconstrained model. Whereas the parameters in the constrained model were restricted to be equal, the parameters in the unconstrained model were allowed to vary freely in the model. All models in the current study were evaluated to determine how well they fit the data. It is often recommended that multiple model fit statistics be used because there are many ways to assess the overall fit of the models with various theoretical frameworks that focus on various aspects of fit (58). The model fit statistics adopted in this study included the relative/normed Chi-square ( $\chi^{2}/df)$ , root mean square error of approximation (RMSEA), standardized root mean squared residual (SRMR), the comparative fit index (CFI), the Tucker-Lewis incremental fit index (TLI), and the goodness-of-fit index (GFI). As recommended by Hu and Bentler (58), an acceptable fit to the data is when ( $\chi^{2}/df)$ < 5; RMSEA and SRMR <0.08; and CFI, TLI, and GFI > 0.90. A comparison of model fits was performed using the Chi-square difference test. While descriptive statistics were estimated with Version 16 of Stata (59), the CFA and SEM models were estimated with SPSS Amos (60).

## 3 Results

### 3.1 Descriptive statistics

Supplementary Table S1 shows detailed information about the sample characteristics of students in HEIs in Sweden in 2021. The proportion of women (63.21%) was higher than that of men (36.79%). The average age for women was higher than that for men (women = 30.92 ± 10.27 versus men = 29.32 ± 9.94).

**Figure 2:**
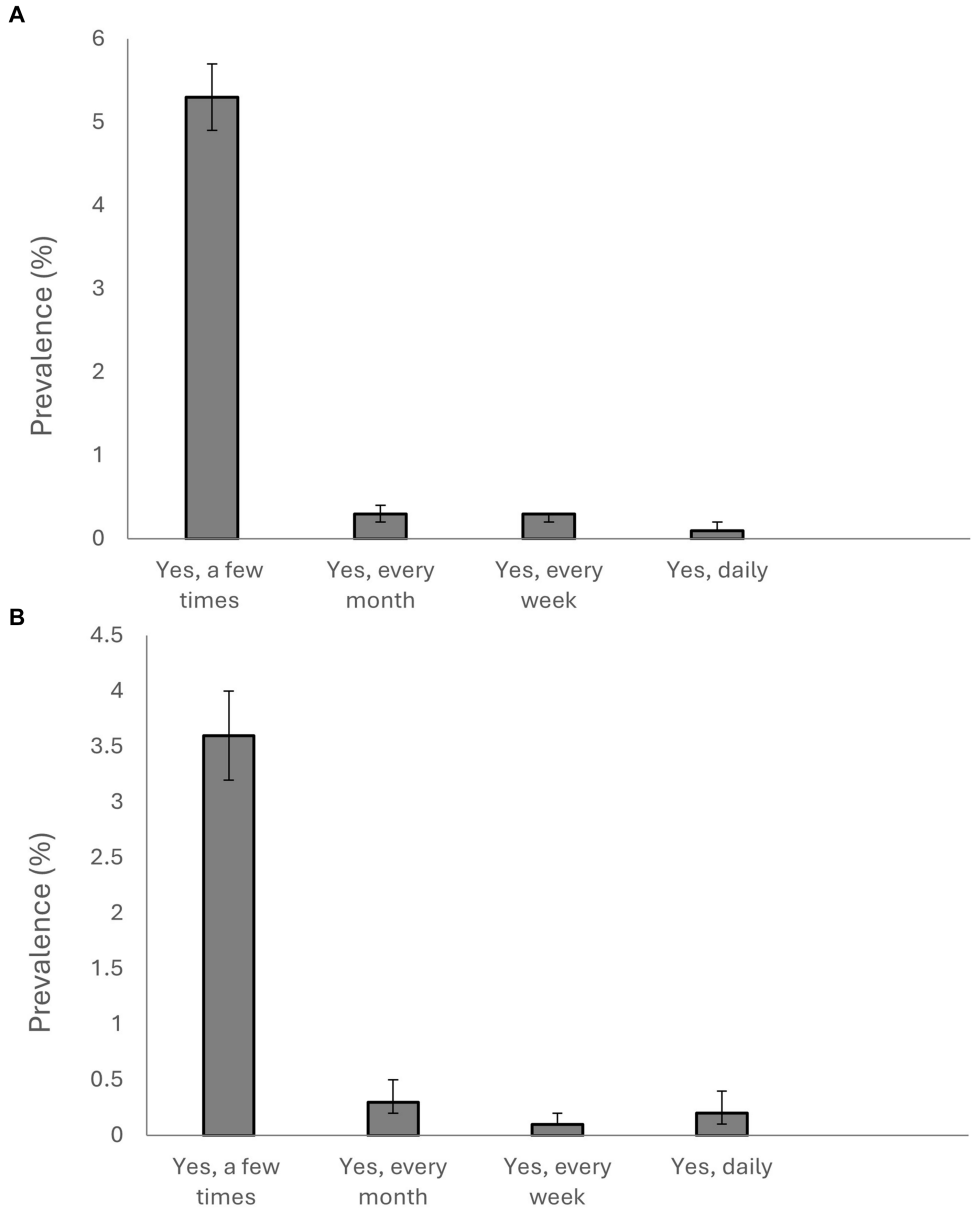
**(A)** Prevalence of bullying among women in higher education institutions in Sweden in 2021. **(B)** Prevalence of bullying among men in higher education institutions in Sweden in 2021.

**Figure 3:**
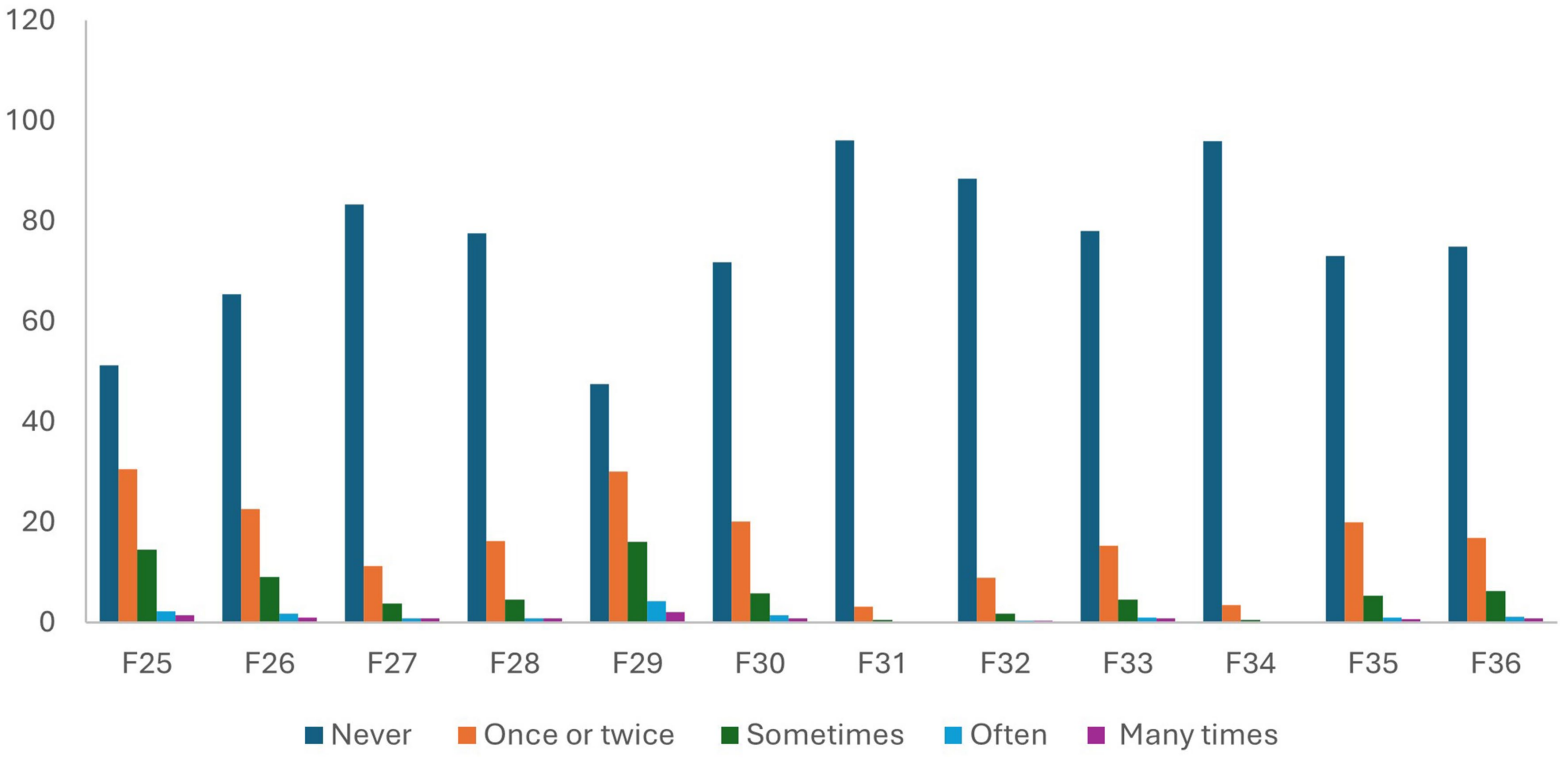
Prevalence of incivility among students in higher education institutions in Sweden in 2021. See Supplementary Table S4 for a description of F25–F36.

The prevalence of incivility, bullying, and health and well-being among students in HEIs in Sweden is presented in Supplementary Tables S2, S3; Figures 2A,B, and 3. The prevalence of students who were bullied a few times was 4.7%, with women being the majority (women = 5.3% versus men = 3.6%). Overall, 5.43% of students indicated that they had experienced bullying in Sweden. The prevalence of bullying among women was higher than among men (women = 6.1% versus men = 4.3%). The results for the items that were used to estimate incivility showed that the most prevalent form of incivility experienced by students was “interrupted or spoke over you” (total population = 52.5%; women = 55% versus men = 48.4%), followed by “paid little attention to your statements or showed little interest in your opinions,” (total population = 48.7%; women = 51.3% versus men = 44.2%). In general, experiencing incivility was more prevalent among women than men. Also, in general, men reported higher proportion of poor health and well-being than women.

### 3.2 Confirmatory factor analysis

Confirmatory factor analysis was performed to establish the reliability and validity of the latent variables (factors) used in the study. Two competing CFA models (Models 1A and 1B) were fitted to determine which measurement model fit the data better. Model 1A was restricted to include all the items that have theoretically and empirically been shown to load on the latent variables. Model modification was performed by employing the modification indices in AMOS. This was accomplished by covarying the error terms of the items in each factor. Table 1 provides detailed information on the measurement models. The goodness-of-fit indices of Model 1A showed an adequate fit to the data. However, two of the twelve items that were used to construct incivility, including “yelled, shouted, or sworn at you?” and “targeted you with anger outbursts or “temper tantrums”?” had unacceptable factor loadings $\left(\lambda <0.50\right)$ . Similarly, one of the eight items that was used to measure poor health and well-being, “have you considered resigning from your current studies?” had a factor loading of λ<0.50 . Thus, items that had low factor loadings on their specific constructs were removed, and a new model (Model 1B) was fitted to the data. The results of Model 1B ( $\chi^{2}/df=$ 4.86, RMSEA = 0.02, SRMR = 0.01, CFI = 0.99, TLI = 0.99, GFI = 0.99) showed a better fit to the data than Model 1A. We then proceeded to estimate the reliability and validity of Model 1B.

**Table 1:** Model fit indices for measurement and structural models of the relationships between incivility, bullying, and poor health and well-being.

Model	$\chi^{2}/df$	RMSEA	SRMR	CFI	TLI	GFI	Comparison	$\Delta \chi^{2}$	Δdf
Model 1A	11.00	0.02	0.03	0.99	0.99	0.99			
Model 1B	4.86	0.02	0.01	0.99	0.99	0.99			
Model 2	27	0.039	0.038	0.98	0.96	0.98			
Unconstrained model	13.89	0.027	0.032	0.98	0.96	0.98			
Constrained model	13.28	0.027	0.032	0.98	0.97	0.98	CM-UM	151***	23

Significance level: *** *p* < 0.01.

### 3.3 Reliability and validity of constructs

The results for the reliability and validity of the latent constructs are presented in Tables 2, 3 and Supplementary Table S5. The Cronbach alpha for incivility ( α=0.88) and poor health and well-being ( α=0.90) showed good internal consistency in terms of reliability. Furthermore, a good composite reliability was obtained for both incivility ( CR=0.89) and poor health and well-being ( CR=0.90) . Thus, the reliability of the latent constructs (i.e., incivility and poor health and well-being) was established in our study. All items that were used to construct incivility and poor health and well-being in Model 1B had acceptable factor loadings $\left(\lambda >0.50\right)$ .

**Table 2:** Internal and construct validity of all latent variables: incivility and poor health and well-being.

Variable/construct	Cronbach alpha	CR	MaxR(H)	AVE	MSV
Incivility	0.88	0.89	0.91	0.46	0.15
PHW	0.90	0.9	0.92	0.575	0.15

**Table 3:** Discriminant validity of all latent variables: incivility and poor health and well-being.

Variable/construct	Incivility	PHW
	√AVE	√AVE
Incivility	0.68	
PHW	(0.39)***	0.76

Significance level: *** *p* <0.01; () is the correlation between constructs; PHW: poor health and well-being.

Also, even though the AVE value for poor health and well-being was acceptable $\left(AVE>0.50\right)$ , the AVE value for incivility did not meet the minimum cut-off of 0.50. Despite the low value of AVE for incivility, the convergent validity of the two constructs may be adequate given that their composite reliability was established in our study. According to Malhotra and Dash (61), convergent validity may be established using only composite reliability because AVE is often too strict.

Lastly, both the incivility and poor health and well-being constructs showed discriminant validity, as their AVEs were greater than their MSVs. This was further confirmed in Table 3, as the square root of the AVEs for both the incivility and poor health and well-being constructs were greater than the correlation between them $\left(\gamma =0.39,P<0.01\right)$ . An additional analysis performed to check the correlation between incivility construct and bullying showed a moderate positive correlation of γ=0.55,P<0.01 .

### 3.4 Direct and indirect effects

**Figure 4:**
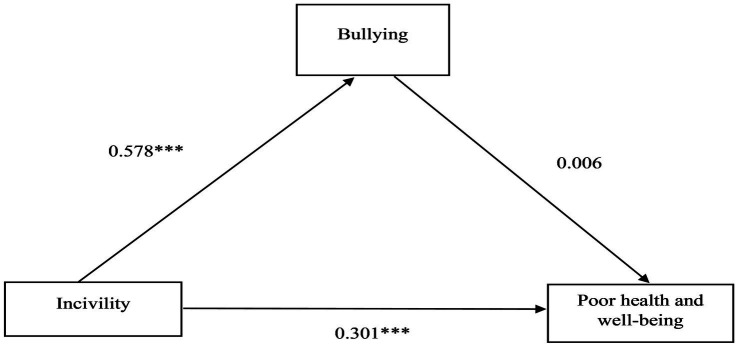
Standardized direct effects, based on SEM, in the relationships between incivility, bullying, and poor health and well-being. Significance level: *** *p* < 0.001.

Structural equation modeling was employed to estimate the direct and indirect relationships between incivility, bullying, and poor health and well-being. To do so, we first determined whether the goodness-of-fit statistics of the mediating effect model (Model 2) significantly fit the data. The details of the goodness-of-fit statistics are presented in Table 1. After adjusting for socio-demographic and educational characteristics, Model 2 demonstrated an acceptable fit to the data ( $\chi^{2}/df=$ 27.00, RMSEA = 0.039, SRMR = 0.035, CFI = 0.98, TLI = 0.96, GFI = 0.98). More specifically, even though the Chi-square fit index did not meet the Hu and Bentler recommended cut-off criteria of $\chi^{2}/df<5$ , the other fit indices showed an excellent fit to the data. Given that the Chi-square is not always the last word in determining the suitability of the model fit, Model 2 was accepted. Thus, we proceeded to estimate the direct and indirect relationships between incivility, bullying, and poor health and well-being. The results are presented in Table 4 and Figure 4. The results of the study showed a positive and direct relationship $\left(\beta =0.301,P<0.01\right)$ between incivility and poor health and well-being, supporting Hypothesis 1. Additionally, we found a positive and direct relationship $\left(\beta =0.578,P<0.01\right)$ between incivility and bullying. This result also supported Hypothesis 2. Contrary to Hypothesis 3, the results did not indicate a significant relationship $\left(\beta =0.006,P>0.05\right)$ between bullying and poor health and well-being. Based on the recommended bias-corrected bootstrapping criteria (57), the results showed an insignificant mediating effect (*β* = 0.003, 95% CI: −0.016–0.008). This suggests that bullying did not significantly mediate the relationship between incivility and poor health and well-being, contrary to our expectations (H4).

**Table 4:** Direct and indirect relationships between incivility, bullying, and poor health and well-being.

Path Analysis	Estimates
Incivility→Bullying	0.578***
Incivility→PHW	0.301***
Bullying→PHW	0.006 NS
Incivility→Bullying→PHW	0.003(−0.016 0.008)

Significance level: ***p < 0.001, NS: not significant; PHW: poor health and well-being.

### 3.5 Gender difference

To understand whether gender influenced the relationship between incivility, bullying, and poor health and well-being among students in HEIs in Sweden, a multigroup analysis was performed. To do so, two models, which were known as the unconstrained and constrained models, were fitted. We first determined whether both models fit the data well. Detailed information on the constrained and unconstrained models is presented in Table 1. Both the unconstrained ( $\chi^{2}/df=$ 13.89, RMSEA = 0.027, SRMR = 0.032, CFI = 0.98, TLI = 0.96, GFI = 0.98) and constrained models ( $\chi^{2}/df=$ 13.28, RMSEA = 0.027, SRMR = 0.032, CFI = 0.98, TLI = 0.97, GFI = 0.98) had an acceptable fit to the data. However, the results of the Chi-square difference test showed that the constrained model fitted the data significantly better than the unconstrained model ( $\Delta \chi^{2}\left(23\right)=179.18,p<0.01)$ , suggesting a significant gender difference in the relationship between incivility, bullying, and poor health and well-being. We subsequently explored each path (H1, H2, and H3) to determine which of the paths was influenced by gender. Table 5, Supplementary Table S6, and Supplementary Figure S1 provide detailed information on the gender difference for each path. The results did not show a significant gender difference in the direct association between incivility and poor health and well-being ( $\Delta \chi^{2}\left(1\right)=1.427,p>0.05)$ , although the magnitude of this particular association was stronger for women $\left(\beta =0.319,\;P<0.01\right)$ than men $\left(\beta =0.300,\;P<0.01\right)$ . Additionally, we found a significant gender difference in the direct association between incivility and bullying ( $\Delta \chi^{2}\left(1\right)=6.41,p<0.05)$ . However, the association was stronger among women $\left(\beta =0.571,\;P<0.01\right)$ than men $\left(\beta =0.545,\;P<0.01\right)$ . Lastly, there was a significant gender difference in the direct relationship between bullying and poor health and well-being ( $\Delta \chi^{2}\left(1\right)=5.629,p<0.05)$ . The relationship was not significant for women but was for men $\left(\beta =0.029,P<0.10\right)$ .

**Table 5:** Direct relationships between incivility, bullying, and poor health and well-being, stratified by gender.

Path Analysis	Estimates	
	Men	Women
Incivility→Bullying	0.545***	0.571***
Incivility→Poor health and well-being	0.300***	0.319***
Bullying→Poor health and well-being	0.029*	0.019

Significance level: *** *p* <0.001, ** *p* < 0.05, * *p* < 0.10.

## 4 Discussion

Using a cross-sectional dataset drawn from 38 HEIs in Sweden, this study investigated the relationships between incivility, bullying, and poor health and well-being and whether there are any gender differences in these associations. The study further explored whether bullying played a mediating role in the nexus between incivility and poor health and well-being among students. Our results show that 3.9–52.5% of students reported various forms of incivility. Also, 5.43% of students experienced bullying. Incivility had a statistically significant and direct relationship with bullying, as well as poor health and well-being. A statistically significant relationship between bullying and poor health and well-being was not found. There were gender differences in these relationships. The relationship between incivility and poor health and well-being via bullying was not significant. To the best of our knowledge, this is the first study to use a large and nationally representative sample to assess the mediating effect of bullying in the relationship between incivility and poor health and well-being among students in HEIs in Sweden.

### 4.1 The relationships between incivility, bullying, and poor health and well-being

Consistent with previous studies (3, 13, 14, 19, 21–23), our study confirmed H1 in that incivility has a direct relationship with poor health and well-being. Although incivility is not considered as an intense form of interpersonal mistreatment, the strong relationship between incivility and poor health and well-being that was observed in this cross-sectional study suggests that the relationship is fairly immediate. One potential explanation for our findings may be attributed to the stressor-strain model (3, 20). The transactional theory of stress and coping posits that daily hassles like incivility (3), which are appraised as threatening over a period, may consequently impair psychosomatic well-being and health (20). Furthermore, our findings corroborated H2 in that there is a strong relationship between incivility and bullying. This result is in line with previous studies (14, 15) that support the escalation model, which proposes that mistreatment with lower intensity may gradually transform into a more intense mistreatment when repeated over a period of time (62). This implies that incivility, which is characterised as a covert act with low intensity and ambiguity, may systematically become overt, unambiguous, and more intense. Another potential reason for our findings is that there may be a bi-directional relationship between incivility and bullying and that those who experience incivility do so because they are already being bullied (14). However, due to the cross-sectional nature of our study design, this type of analysis is beyond the scope of our research.

Regarding H3, the relationship between bullying and poor health and well-being was not statistically significant. This particular result is inconsistent with previous research, which has shown that bullying has a direct relationship with negative health outcomes (31, 33, 34). For example, a recent meta-analysis that was conducted on longitudinal studies established that bullying, in terms of peer victimization, may predict future suicidal ideation (34). Similarly, a cross-sectional study conducted among university students in Germany and China concluded that bullying experiences are associated with depression, anxiety, stress symptoms, and poor emotional well-being and health (63). Nevertheless, unlike our study, the operationalisation of bullying in Lin et al. (63) and the majority of the longitudinal studies in the meta-analysis presented in Van Geel et al. (34) were based on a multiple-item questionnaire. Several factors may explain our unexpected findings. First, the low prevalence of bullying (5.4%) among students in Sweden may explain why the relationship between bullying and poor health and well-being was not significant. Secondly, the use of a single-item questionnaire may not have adequately captured a complex construct like bullying, and this may have contributed to the low prevalence and statistical power (64).

We expected bullying to play a mediating role in the relationship between incivility and poor health and well-being. Contrary to our expectations (H4), the mediating effect of bullying was not statistically significant. Because this finding is novel, we were unable to compare our results with those of previous studies. Our findings should be taken with caution because the occurrence of bullying among students may have decreased given the hybrid learning policy that was in place in Sweden in 2021. The hybrid policy was implemented to fight the spread of the COVID-19 pandemic in Sweden. A study that compared the prevalence of bullying among students before and during the COVID-19 pandemic demonstrated a higher rate of bullying in all forms (general, physical, social, and verbal), except cyberbullying, before the COVID-19 pandemic as compared to during the COVID-19 pandemic (65).

### 4.2 Gender differences in the relationships

Given that the multigroup analysis revealed a significant gender difference in the model of the relationships between incivility, bullying, and poor health and well-being, we proceeded to estimate for the gender difference for each path. Previous findings on gender differences in the relationship between incivility and negative health outcomes have been mixed. While some studies have identified a stronger relationship for women (44), other studies have found a stronger relationship for men (13). In this study, we found that the relationship between incivility and poor health and well-being did not differ between women and men, which was contrary to previous studies (13, 44). This finding is very notable, especially given that the prevalence of forms of incivility among women in this study was comparatively higher than that for men. Such findings highlight women’s strength and resilience (66). Lim et al. (66) demonstrated that even though women experience higher levels of hostile treatment in organisations than men, they do not feel more stress, are less likely to quit their jobs, and are more satisfied with their jobs than men.

This study illustrates a stronger association between incivility and bullying for women than men. One possible reason for this finding is the higher prevalence of incivility that was reported among women than men in this study. This finding is in line with previous studies that also examined gender differences in incivility (1, 38, 41). Social role theory posits that gender norms expect men to show more agency-based traits (e.g., aggressiveness and dominance) than women, and women, on the other hand, are expected to show more communal-based traits (e.g., nurturing, compassion, and cooperativeness) than men (37). As men and women are expected to conform to gender norms to maintain good social standing, they may also receive backlash when they violate gender norms. Meanwhile, because women are often expected to be more communal than men, they may be more likely to become targets of incivility (1, 38, 41), and as mentioned above, this may subsequently escalate to more intense behavior, such as bullying (14–16).

Lastly, even though there was no statistically significant relationship between bullying and poor health and well-being among women, a weak relationship was found for men. Again, according to social role theory, the expectation that women should possess more communal-based traits (e.g., nurturing, compassion, and cooperativeness) than men, while men should possess more agency-based traits (e.g., aggressiveness and dominance) than women (37), makes women more vulnerable to bullying (39, 42). A similar finding was revealed in our study, as more women reported being targets of bullying as compared to men. Despite this result, men had associated poor health and well-being, but not women, which was contrary to previous studies (43, 45). Our findings could be explained by the expectation violation theory, which indicates that individuals react more negatively when their expectations are unmet (67). Because men are often the perpetrators and not the targets of bullying, they may not expect this exposure; hence, a violation of this expectation may have a more detrimental health outcome as compared to women, who may be used to such acts. Another possible reason for this finding may be attributed to women’s strength and resilience (66). Because women are more likely to experience bullying than men, women may perhaps build more coping skills, which in turn may have a weaker association with negative health outcomes than men.

### 4.3 Strengths, limitations, and future studies

The use of a sizable and nationally representative sample of students from 38 HEIs in Sweden is a key strength of the current study. Nonetheless, it should be noted that women, older students, and students born in Sweden are overrepresented in the current sample. Another major strength of this study is that we expanded the literature by using a cross-sectional study design to provide empirical and novel findings regarding the direct relationship between incivility and bullying among students in HEIs in Sweden. This adds to the existing literature, which has only used a longitudinal study design and a working population in Sweden (14). The current study also contributed to the literature on the effect of gender in the relationships between incivility, bullying, and poor health and well-being in students. Furthermore, this study provided new insight indicating that the pathway between incivility and poor health and well-being among students in higher education was not significantly mediated by bullying. However, this finding should be taken with caution, as the few cases of bullying in HEIs in Sweden during the COVID-19 pandemic may have influenced this outcome. Another key strength is that the use of CFA in this study provided reliability and construct validity for the 12-item incivility scale, which have never been reported before for student populations in higher education settings in Sweden. Lastly, the application of SEM with maximum likelihood estimation in this study produced robust findings.

Despite these strengths, this study has a few limitations. First, operationalisation of the item bullying in the COPSOQ III questionnaire may be ambiguous, as using the word “have you been exposed to bullying at your place of study” could mean either being bullied yourself or witnessing bullying or both. Furthermore, the use of a single-item question to measure bullying, which does not distinguish between physical bullying and cyberbullying, may have affected the results. Additionally, a single-item measure of bullying does not include other aspects of information such as the perpetrators, severity, and position. However, a single-item measure of bullying is frequently utilized in research (64) and has been identified to predict negative health outcome (68). We recommend that future studies should use a multiple-item questionnaire to adequately capture bullying in all forms (general, physical, social, verbal, and cyber) (65). We also recommend that the bullying item in COPSOQ III should be accompanied with detailed explanation for respondents to understand whether the intent for the question is to capture being bullied or witnessing bullying or both. Second, the hybrid-learning program that was in force during the data collection process, where teaching and administration were conducted both online and in person, could have affected our results. This is because, available evidence suggests that interpersonal mistreatment, such as bullying, in organizations reduced during the COVID-19 pandemic (65). Nevertheless, our findings did not differ significantly from previous results. We recommend that future studies be conducted post-pandemic to understand how mistreatment, like incivility and bullying, has changed in HEIs since the COVID recovery, when teaching and administration began to be mostly conducted in person again. Third, due to the cross-sectional design, this study did not establish causality for the hypotheses proposed. There is always a possibility of reverse causation. We recommend that future studies replicate this study with a longitudinal design.

### 4.4 Practical implications

This study underscores several practical implications. Considering our overall findings, overt, unambiguous, and intense behavior, like bullying, persists among students in Sweden, but its prevalence is low. On the other hand, covert and less intense behavior, like incivility, has a high prevalence. Meanwhile, just like bullying, incivility has detrimental health and well-being outcomes. Thus, this study highlights the need to design and develop effective strategies for reducing incivility and bullying in HEIs. Government and university authorities must ensure that policies and regulations that target the reduction of interpersonal mistreatment, e.g., the Swedish higher education ordinance (12), should not only include bullying but should also focus on incivility. University authorities should ensure that they create a hospitable and friendly culture in the school environment to help students and faculty members treat one another with respect, decency, civility, and fairness. Additionally, they should ensure that every student is aware of the code of conduct that guides student behavior in the school. This could be done by including it in the syllabus or through the organization of workshops and seminars. The code of conduct should explicitly state what behaviors are considered uncivil and bullying to help students easily appraise and report such acts. Governments and university authorities must offer counseling sections not only to students who are victims of bullying but also victims of incivility, as well as bystanders, to help improve the general health and well-being of students. Additionally, there should be more avenues for students who are victims or bystanders to easily report these kinds of mistreatment and stand-up to the perpetrators. Governments, university authorities, and policymakers must consider gender differences in incivility and bullying when developing policies and interventions that reduce these kinds of behaviors in organizations.

## 5 Conclusion

This study demonstrated relationships between incivility and bullying, as well as poor health and well-being among students in higher education institutions in Sweden. Moreover, gender accounted for a significant difference in these relationships. Even if our findings imply that bullying did not play a significant mediating role in the link between incivility and poor health and well-being, bullying remains an important factor to consider in the nomological network between incivility and poor health and well-being. Further work is needed to investigate this relationship, preferably using longitudinal study designs and in different settings. Our findings underscore the need to consider gender differences when designing and developing effective policies, strategies, and interventions aimed at reducing incivility and bullying, as well as promoting good health and well-being in higher education institutions.

## Data Availability

The raw data supporting the conclusions of this article will be made available by the authors, without undue reservation.
