# Supplementary material for: Incivility, bullying, and poor health and well-being among students: a Swedish national study in higher education institutions
Source: Front Public Health. 2024 Oct 30;12:1400520. doi: 10.3389/fpubh.2024.1400520 (PMC11557468; doi:10.3389/fpubh.2024.1400520)
Supplement: Supplementary file 1 [file Data_Sheet_1.docx]

Appendix 1

Table S1 General characteristics of students in higher education institutions in Sweden in 2021

| Variables | Men | Women |
| --- | --- | --- |
|  | % or mean±SD | % or mean±SD |
| **Age** | 29.32±9.94 | 30.92±10.27 |
| **Birthplace** |  |  |
| Not born in Sweden | 12.24% | 13.59% |
| Born in Sweden, with two parents not born in Sweden | 4.96% | 5.16% |
| Born in Sweden, with one parent not born in Sweden | 10.19% | 9.35% |
| Born in Sweden, with both parents born in Sweden | 72.61% | 71.89% |
| **Subjects** |  |  |
| Science | 16.06% | 9.33% |
| Engineering | 27.59% | 7.79% |
| Medicine and health sciences | 11.90% | 22.93% |
| Agricultural and veterinary sciences | 1.43% | 2.79% |
| Social sciences | 29.17% | 42.68% |
| Humanities and arts | 12.01% | 13.63% |
| Others | 1.85% | 0.86% |

Table S2 Prevalence of bullying and health and well-being among students in higher education institutions in Sweden in 2021

| Variable | Total Pop |  | Men |  | Women |  |
| --- | --- | --- | --- | --- | --- | --- |
|  | Prev (%) | CI | Prev (%) | CI | Prev (%) | CI |
| ***Bullying*** |  |  |  |  |  |  |
| No | 94.6 | 94.2–94.9 | 95.6 | 95.1–96.1 | 93.9 | 93.4–94.3 |
| Yes, a few times | 4.7 | 4.4–5.0 | 3.6 | 3.2–4.0 | 5.3 | 4.9–5.7 |
| Yes, every month | 0.3 | 0.2–0.4 | 0.3 | 0.2–0.5 | 0.3 | 0.2–0.4 |
| Yes, every week | 0.2 | 0.1–0.3 | 0.1 | 0.1–0.2 | 0.3 | 0.2–0.3 |
| Yes, daily | 0.2 | 0.1–0.2 | 0.2 | 0.1–0.4 | 0.1 | 0.1–0.2 |
| ***Health and well-being*** |  |  |  |  |  |  |
| **Had problem relaxing** |  |  |  |  |  |  |
| Not at all | 14.6 | 14.1–15.1 | 5.2 | 4.7–5.8 | 7.5 | 7.0–8.0 |
| A small part of the time | 29.1 | 28.5–29.8 | 18.2 | 17.2–19.1 | 24.9 | 24.1–25.7 |
| Part of the time | 27.2 | 26.5–27.9 | 25.3 | 24.3–26.5 | 28.3 | 27.4–29.1 |
| A large part of the time | 22.4 | 21.8–23.1 | 32.7 | 31.6–33.8 | 27.0 | 26.2–27.9 |
| All the time | 6.6 | 6.3–7.0 | 18.5 | 17.5–19.4 | 12.3 | 11.7–12.9 |
| **Been irritatable** |  |  |  |  |  |  |
| Not at all | 17.9 | 17.4–18.5 | 2.0 | 1.7–2.4 | 4.3 | 4.0–4.7 |
| A small part of the time | 35.8 | 35.1–36.5 | 9.5 | 8.8–10.2 | 16.9 | 16.2–17.6 |
| Part of the time | 28.6 | 28.0–29.3 | 24.4 | 23.4–25.5 | 31.1 | 30.2–32.0 |
| A large part of the time | 14.2 | 13.7–14.7 | 39.4 | 38.2–40.6 | 33.7 | 32.8–34.6 |
| All the time | 3.5 | 3.2–3.7 | 24.7 | 23.7–25.8 | 14.0 | 13.3–14.6 |
| **Been tensed** |  |  |  |  |  |  |
| Not at all | 16.0 | 15.5–16.6 | 3.1 | 2.7–3.5 | 5.2 | 4.8–5.7 |
| A small part of the time | 29.4 | 28.7–30.1 | 16.6 | 15.8–17.6 | 23.6 | 22.8–24.4 |
| Part of the time | 29.1 | 28.4–29.8 | 27.5 | 26.5–28.6 | 30 | 29.2–30.9 |
| A large part of the time | 21.0 | 20.4–21.6 | 31.5 | 30.4–32.7 | 28.1 | 27.3–29.0 |
| All the time | 4.4 | 4.1–4.7 | 21.2 | 20.3–22.2 | 13.0 | 12.4–13.7 |
| **Felft worn out** |  |  |  |  |  |  |
| Not at all | 11.8 | 11.3–12.2 | 6.5 | 6.0–7.1 | 9.3 | 8.8–9.9 |
| A small part of the time | 28.2 | 27.6–28.9 | 20.0 | 19.0–21.0 | 25.9 | 25.1–26.7 |
| Part of the time | 28.0 | 27.3–28.6 | 28.6 | 27.5–29.7 | 27.6 | 26.8–28.4 |
| A large part of the time | 23.7 | 23.1–24.3 | 30.0 | 28.9–31.1 | 27.2 | 26.4–28.1 |
| All the time | 8.3 | 7.9–8.7 | 14.9 | 14.1–15.8 | 10.0 | 9.4–10.5 |
| **Been physically exhausted** |  |  |  |  |  |  |
| Not at all | 23.6 | 22.9–24.2 | 2.2 | 1.9–2.6 | 4.6 | 4.3–5.0 |
| A small part of the time | 33.5 | 32.8–34.2 | 10.3 | 9.6–11.0 | 15.6 | 15.0–16.3 |
| Part of the time | 25.5 | 24.9–26.2 | 25.1 | 24.1–26.2 | 25.8 | 25.0–26.6 |
| A large part of the time | 13.7 | 13.1–14.2 | 35.0 | 33.8–36.1 | 32.7 | 31.8–33.5 |
| All the time | 3.7 | 3.5–4.0 | 27.4 | 26.3–28.5 | 21.3 | 20.5–22.1 |
| **Been emotionally exhausted** |  |  |  |  |  |  |
| Not at all | 20.9 | 20.3–21.5 | 4.9 | 4.4–5.4 | 10.3 | 9.8–10.9 |
| A small part of the time | 26.2 | 25.5–26.8 | 15.2 | 14.4–16.1 | 23.4 | 22.6–24.2 |
| Part of the time | 24.3 | 23.6–24.9 | 21.4 | 20.4–22.4 | 25.9 | 25.1–26.7 |
| A large part of the time | 20.4 | 19.8–21.0 | 29.3 | 28.2–30.4 | 24.3 | 23.5–25.1 |
| All the time | 8.3 | 7.9–8.7 | 29.1 | 28.0–30.3 | 16.1 | 15.4–16.8 |
| **In general, would you say your health is** |  |  |  |  |  |  |
| Excellent | 10.8 | 10.4–11.3 | 14.9 | 14.1–15.8 | 8.4 | 7.9–8.9 |
| Very good | 33.1 | 32.4–23.8 | 34.0 | 32.9–35.2 | 32.5 | 31.7–33.4 |
| Good | 37.6 | 36.9–38.3 | 33.7 | 32.5–34.8 | 39.9 | 38.9–40.8 |
| Fair | 15.5 | 14.9–16.0 | 14.5 | 13.7–15.4 | 16 | 15.4–16.7 |
| Poor | 3.1 | 2.8–3.3 | 2.8 | 2.5–3.3 | 3.2 | 2.9–3.5 |
| **Have you considered resigning from your current studies** |  |  |  |  |  |  |
| Never | 48.2 | 47.5–48.9 | 46.4 | 45.2–47.7 | 49.2 | 48.3–50.2 |
| Once or twice | 29.1 | 28.4–29.7 | 30.0 | 28.9–31.1 | 28.5 | 27.7–29.3 |
| Sometimes | 14.3 | 13.8–14.8 | 14.7 | 13.8–15.5 | 14.1 | 13.5–14.8 |
| Often | 3.7 | 3.4–4.0 | 3.8 | 3.3–4.3 | 3.6 | 3.3–4.0 |
| Many times | 4.7 | 4.4–5.0 | 5.1 | 4.6–5.7 | 4.5 | 4.1–4.9 |

Table S3 Prevalence of incivility among students in higher education institutions in Sweden in 2021

| Variable | Total Pop |  | Men |  | Women |  |
| --- | --- | --- | --- | --- | --- | --- |
|  | Prev (%) | CI | Prev (%) | CI | Prev (%) | CI |
| F25 |  |  |  |  |  |  |
| Never | 51.3 | 50.6–52.1 | 55.8 | 54.6–57.0 | 48.7 | 47.7–49.6 |
| Once or twice | 30.5 | 29.8–31.1 | 28.1 | 27.0–29.1 | 31.8 | 30.9–32.7 |
| Sometimes | 14.6 | 14.08-15.1 | 12.9 | 12.1–13.8 | 15.5 | 14.9–16.2 |
| Often | 2.2 | 2.1–2.5 | 1.7 | 1.4–2.0 | 2.5 | 2.2-.2.8 |
| Many times | 1.4 | 1.2–1.5 | 1.3 | 1.1–1.7 | 1.3 | 1.1–1.6 |
| F26 |  |  |  |  |  |  |
| Never | 65.5 | 64.8–66.2 | 66.5 | 65.4–67.7 | 64.9 | 64.0–65.8 |
| Once or twice | 22.7 | 22.1–23.3 | 22.3 | 21.4–23.3 | 22.9 | 22.1–23.7 |
| Sometimes | 9.1 | 8.7–9.5 | 8.6 | 7.9–9.2 | 9.3 | 8.8–9.9 |
| Often | 1.7 | 1.5–1.9 | 1.5 | 1.2–1.8 | 1.7 | 1.5–2.0 |
| Many times | 1 | 0.82–1.1 | 0.9 | 0.7–1.1 | 0.9 | 0.8–1.2 |
| F27 |  |  |  |  |  |  |
| Never | 83.4 | 82.9–84.0 | 86.5 | 85.6–87.3 | 81.6 | 80.9–82.3 |
| Once or twice | 11.2 | 10.7–11.6 | 9 | 8.3–9.7 | 12.4 | 11.8–13.0 |
| Sometimes | 3.8 | 3.5–4.1 | 3 | 2.67–3.5 | 4.2 | 3.8–4.5 |
| Often | 0.9 | 0.8–1.0 | 0.7 | 0.5–0.9 | 0.9 | 0.8–1.2 |
| Many times | 0.8 | 0.63–0.89 | 0.6 | 0.4–0.8 | 0.8 | 0.6–0.9 |
| F28 |  |  |  |  |  |  |
| Never | 77.5 | 76.9–78.1 | 80 | 79.0–80.9 | 76.1 | 75.2–76.8 |
| Once or twice | 16.3 | 15.8–16.9 | 13.7 | 12.9–14.5 | 17.8 | 17.1–18.6 |
| Sometimes | 4.6 | 4.3–4.9 | 4.5 | 4.0–5.0 | 4.5 | 4.2–5.0 |
| Often | 0.8 | 0.7–0.9 | 0.7 | 0.5–0.9 | 0.8 | 0.6–1.0 |
| Many times | 0.8 | 0.7–0.9 | 1 | 0.8–1.3 | 0.6 | 0.5–0.8 |
| F29 |  |  |  |  |  |  |
| Never | 47.5 | 46.8–48.3 | 51.6 | 50.4–52.9 | 45 | 44.1–46.0 |
| Once or twice | 30.1 | 29.4–30.8 | 29.2 | 28.1–30.3 | 30.6 | 29.7–31.4 |
| Sometimes | 16.1 | 15.6–16.7 | 14.6 | 13.8–15.5 | 16.9 | 16.2–17.6 |
| Often | 4.3 | 4.0–4.6 | 2.8 | 2.5–3.3 | 5 | 4.7–5.5 |
| Many times | 2 | 1.8–2.2 | 1.5 | 1.2–1.9 | 2.2 | 2.0–2.5 |
| F30 |  |  |  |  |  |  |
| Never | 71.8 | 71.1–72.5 | 72.2 | 71.1–73.3 | 71.5 | 70.6–72.3 |
| Once or twice | 20.2 | 19.6–20.8 | 19.6 | 18.7–20.6 | 20.4 | 19.7–21.2 |
| Sometimes | 5.8 | 5.4–6.1 | 5.7 | 5.2–6.3 | 5.7 | 5.3–6.2 |
| Often | 1.4 | 1.2–1.6 | 1.3 | 1.1–1.6 | 1.4 | 1.2–1.7 |
| Many times | 0.9 | 0.76–1.0 | 0.9 | 0.7–1.1 | 0.8 | 0.6–1.0 |
| F31 |  |  |  |  |  |  |
| Never | 96.1 | 95.8–96.3 | 94.8 | 94.2–95.3 | 96.7 | 96.4–97.0 |
| Once or twice | 3.1 | 2.9–3.4 | 3.9 | 3.4–4.4 | 2.6 | 2.3–2.9 |
| Sometimes | 0.5 | 0.4–0.6 | 0.7 | 0.5–0.9 | 0.3 | 0.2-.0.5 |
| Often | 0.1 | 0.1–0.2 | 0.1 | 0.1–0.3 | 0.1 | 0.1–0.2 |
| Many times | 0.2 | 0.1–0.3 | 0.2 | 0.1–0.4 | 0.1 | 0.1–0.2 |
| F32 |  |  |  |  |  |  |
| Never | 88.5 | 88.0–89.0 | 89.4 | 88.6–90.1 | 87.9 | 87.3–88.5 |
| Once or twice | 9 | 8.5–9.4 | 8.2 | 7.5–8.8 | 9.4 | 8.8–9.9 |
| Sometimes | 1.8 | 1.6–2.0 | 1.7 | 1.4–2.0 | 1.8 | 1.6–2.1 |
| Often | 0.4 | 0.3–0.5 | 0.3 | 0.1–0.4 | 0.4 | 0.3–0.5 |
| Many times | 0.3 | 0.3–0.4 | 0.4 | 0.2–0.5 | 0.3 | 0.2–0.4 |
| F33 |  |  |  |  |  |  |
| Never | 78.1 | 77.5–78.7 | 80 | 79.0–81.0 | 76.9 | 76.1–77.7 |
| Once or twice | 15.3 | 14.8–15.9 | 14.2 | 13.4–15.1 | 15.9 | 15.3–16.6 |
| Sometimes | 4.6 | 4.3–5.0 | 4.1 | 3.6–4.6 | 4.9 | 4.5–5.3 |
| Often | 1 | 0.9–1.2 | 0.8 | 0.6–1.0 | 1.1 | 1.0–1.3 |
| Many times | 0.9 | 0.8–1.0 | 0.7 | 0.5–1.0 | 0.9 | 0.8–1.1 |
| F34 |  |  |  |  |  |  |
| Never | 95.8 | 95.5–96.1 | 95.9 | 95.4–96.4 | 95.7 | 95.2–96.0 |
| Once or twice | 3.5 | 3.2–.3.8 | 3.2 | 2.8–3.6 | 3.6 | 3.2–4.0 |
| Sometimes | 0.5 | 0.4–0.6 | 0.5 | 0.3–0.7 | 0.5 | 0.3–0.6 |
| Often | 0.1 | 0.0–0.1 | 0.1 | 0.0–0.2 | 0.1 | 0.0–0.1 |
| Many times | 0.2 | 0.1–0.3 | 0.2 | 0.1–0.3 | 0.1 | 0.1–0.2 |
| F35 |  |  |  |  |  |  |
| Never | 73 | 72.3–73.6 | 74.7 | 73.6–75.7 | 71.9 | 71.1–72.7 |
| Once or twice | 20 | 19.4–20.6 | 18.9 | 18.0–19.9 | 20.6 | 19.9-21.41 |
| Sometimes | 5.3 | 5.0–5.6 | 4.8 | 4.2–5.3 | 5.6 | 5.2–6.0 |
| Often | 1 | 0.8–1.1 | 0.8 | 0.7–1.1 | 1 | 0.8–1.2 |
| Many times | 0.7 | 0.6–0.8 | 0.6 | 0.5–0.9 | 0.7 | 0.5–0.9 |
| F36 |  |  |  |  |  |  |
| Never | 74.9 | 74.2–75.5 | 69.5 | 68.3–70.6 | 78 | 77-.78.9 |
| Once or twice | 16.9 | 16.3–17.4 | 19.1 | 18.1–20.0 | 15.5 | 14.9 16.2 |
| Sometimes | 6.3 | 6.0–6.7 | 8.4 | 7.7–9.2 | 5.1 | 4.7–5.4 |
| Often | 1.2 | 1.0–1.4 | 1.8 | 1.5–2.1 | 0.8 | 0.6–1.0 |
| Many times | 0.8 | 0.6–0.9 | 1.1 | 0.8–1.4 | 0.5 | 0.4–0.6 |

Table S4 Description of incivility items

| Items | Question |
| --- | --- |
| F25 | Has someone paid little attention to your statements or showed little interest in your opinions? |
| F26 | Doubted your judgement on a matter over which you had responsibility? |
| F27 | Given you hostile looks, stares, or sneers? |
| F28 | Addressed you in unprofessional terms, either publicly or privately? |
| F29 | Interrupted or “spoke over” you? |
| F30 | Rated you lower than you deserve on an evaluation? |
| F31 | Yelled, shouted, or sworn at you? |
| F32 | Made insulting or disrespectful remarks about you? |
| F33 | Ignored you or failed to speak to you? |
| F34 | Targeted you with anger outbursts or “temper tantrums?” |
| F35 | Accused you of incompetence? |
| F36 | Made jokes at your expense? |

Table S5 Standardized factor loadings of all items for each latent variable: incivility and poor health and well-being

| Variable/Construct | Items | Standardized factor loading |
| --- | --- | --- |
| Incivility | Paid little attention to your statements or showed little interest in your opinions | 0.82*** |
|  | Doubted your judgement on a matter over which you had responsibility | 0.71*** |
|  | Gave you hostile looks, stares, or sneers | 0.69*** |
|  | Addressed you in unprofessional terms, either publicly or privately | 0.6*** |
|  | Interrupted or “spoke over” you | 0.8*** |
|  | Rated you lower than you deserved on an evaluation | 0.59*** |
|  | Made insulting or disrespectful remarks about you | 0.54*** |
|  | Ignored you or failed to speak to you | 0.71*** |
|  | Accused you of incompetence | 0.69*** |
|  | Made jokes at your expense | 0.57*** |
|  |  |  |
| Poor health and well-being | Had problems relaxing | 0.78*** |
|  | Been irritable | 0.75*** |
|  | Been tense | 0.76*** |
|  | Felt worn out | 0.8*** |
|  | Been physically exhausted | 0.73*** |
|  | Been emotionally exhausted | 0.87*** |
|  | In general, would you say your health is | 0.6*** |

Notes: Significance level: $***p<0.01$

Table S6 Model fit indices for the gender difference in the relationships between incivility, bullying, and poor health and well-being

| Model | $\chi^{2}/df$ | RMSEA | SRMR | CFI | TLI | GFI | ${\Delta\chi}^{2}$ | $\Delta$df |
| --- | --- | --- | --- | --- | --- | --- | --- | --- |
| Unconstraint (UM) | 13.89 | 0.027 | 0.032 | 0.98 | 0.96 | 0.98 |  |  |
| $Inciv*GD$→ *Bul* | 13.86 | 0.027 | 0.032 | 0.978 | 0.964 | 0.981 | 6.41** | 1 |
| $Inciv*GD$→ *P*$HW$ | 13.83 | 0.027 | 0.032 | 0.978 | 0.964 | 0.981 | 1.427 n.s. | 1 |
| $Bul*GD$→ *P*$HW$ | 13.86 | 0.027 | 0.032 | 0.978 | 0.964 | 0.981 | 5.629** | 1 |

Significance level: $***p<0.001$, $**<0.05$, $*<0.10$; Inciv: incivility; Bul: Bullying; PHW: poor health and well-being; GD: gender; NS: not significant

Figure S1 Direct relationships between incivility, bullying, and poor health and well-being, stratified by gender
